# HLA-E expression and its clinical relevance in human renal cell carcinoma

**DOI:** 10.18632/oncotarget.11744

**Published:** 2016-08-31

**Authors:** Barbara Seliger, Simon Jasinski-Bergner, Dagmar Quandt, Christine Stoehr, Juergen Bukur, Sven Wach, Wolfgang Legal, Helge Taubert, Bernd Wullich, Arndt Hartmann

**Affiliations:** ^1^ Institute of Medical Immunology, Martin-Luther-University, Halle-Wittenberg, Germany; ^2^ Institute of Pathology, Friedrich-Alexander-University, Erlangen-Nuremberg, Germany; ^3^ Clinics for Urology, Friedrich-Alexander-University, Erlangen-Nuremberg, Germany

**Keywords:** HLA-E, non-classical HLA class I, renal cell carcinoma, tumor progression, immune escape

## Abstract

The non-classical human leukocyte antigen E (HLA-E) expression is frequently overexpressed in tumor diseases, transplants and virus-infected cells and represents an immunomodulatory molecule by binding to the receptors CD94/NKG2A, -B and –C on NK and T cells. Due to its immune suppressive features HLA-E expression might represent an important mechanism of tumors to escape immune surveillance.

While an aberrant expression of the non-classical HLA-G antigen in human renal cell carcinoma (RCC) has been demonstrated to be associated with a worse outcome of patients and reduced sensitivity to immune effector cell-mediated cytotoxicity, the expression and function of HLA-E has not yet been analyzed in this tumor entity.

Higher levels of HLA-E transcripts were detected in all RCC cell lines and tumor lesions, which were tested in comparison to normal kidney epithelium. Immunohistochemical staining of a tissue microarray (TMA) using the HLA-E-specific monoclonal antibody TFL-033 recognizing the cytoplasmic HLA-E α-chain as monomer revealed a heterogeneous HLA-E expression in RCC lesions with the highest frequency in chromophobe RCC when compared to other RCC subtypes. HLA-E expression did not correlate with the frequency of CD3^+^, CD4^+^, CD8^+^ and FoxP3^+^ immune cell infiltrations, but showed an inverse correlation with infiltrating CD56^+^ cells. In contrast to HLA-G, HLA-E expression in RCCs was not statistically significant associated with a decreased disease specific survival. These data suggest that HLA-E overexpression frequently occurs in RCC and correlates with reduced immunogenicity.

## INTRODUCTION

Tumors have developed different strategies to escape immune surveillance [1], including down-regulation of classical MHC class I antigens and/or components of the antigen processing machinery (APM) [2] or up-regulation of the non-classical HLA-G and –E antigens [3]. While HLA-G binds to the inhibitory receptors ILT2, ILT4 and KIR2DL4, HLA-E is the major ligand for the inhibitory NK cell receptor CD94/NKG2A,- B and for the activating -C expressed on NK cells and cytotoxic T lymphocytes (CTL) [4, 5] resulting in a reduced immune effector cell mediated cytotoxicity [4, 6]. In contrast to HLA-G, HLA-E lacks the existence of soluble splice variants, but both molecules can be shedded from the cellular membrane [7, 8].

The non-classical HLA-E mRNA is detected in all nucleated cells, while HLA-E protein is only ubiquitously expressed at low levels on the cell surface of most tissues and presents peptides derived from the conserved leader sequences of HLA-A, - B, -C and –G antigens to HLA-E-restricted immune effector cells demonstrating its importance for anti-tumor response [4, 9, 10].

Under pathophysiologic conditions, HLA-E expression has been found at a high frequency in a number of human solid and hematopoietic tumors, which often correlated with the presence of the HLA-A*02 class I allele [11, 12]. There exists also evidence that HLA-E expression could be upregulated by HLA-G [13] resulting in an enhanced immune suppression.

The increased HLA-E expression has clinical relevance, since it could be correlated to tumor progression, metastasis formation and reduced survival of patients in some tumor entities, e.g. in laryngeal carcinoma [14], mammary carcinoma [15], small cell lung cancer [16], pancreatic cancer [17] ovarian carcinoma [11] and in colorectal carcinoma [18, 19]. Concerning hematopoietic malignancies HLA-E surface expression was found in a high percentage of lymphoid tumor cells and was associated with a protection from NK cell-mediated cytotoxicity [20]. This was in line with the impaired CD94/NKG2A-dependent NK cell-mediated cytolysis of interferon (IFN)-γ-treated acute myeloid leukemia (AML) cells by NK cells [21] due to the IFN-γ-mediated upregulation of HLA-E on the cell surface.

Until now the antibodies commonly used for monitoring HLA-E expression were 3D12 or MEM-E/02 [22] antibodies, respectively detecting HLA-E in both soluble extracts and/or the cell surface with a low frequency, but unspecifically cross-react with other HLA class Ia antigens. The monoclonal antibody (mAb) TFL-033 has a unique specificity and selectivity, which allows differentiating between the trimeric complex consisting of the HLA-E α-chain, the presented and loaded peptide and β_2_-m microglobulin (β_2_-m) and the β_2_-m-free, unloaded HLA-E monomer [23]. The diagnostic potential of the TFL-033 mAb has been recently described by analyzing HLA-E expression in normal gastric mucosa, primary as well as metastatic gastric cancer [24]. In addition, novel anti-HLA-E mAbs not only allow reliable immunodiagnostics, but also immune modulation of HLA-E and upregulation of CD8^+^ CTL [25].

Renal cell carcinoma (RCC) represents a heterogeneous malignancy with clear cell RCC (ccRCC) as the most frequent type, and in addition with lower frequencies chromophobe and papillary RCC. The correlation of high T cell infiltration with a worse prognosis in RCC and the limited response to T cell-based immunotherapies might be explained by the high frequency of HLA-G expression in RCC lesions [10, 26, 27]. In addition, increased HLA-E mRNA transcript levels in RCC lesions were associated with a worse prognosis of RCC patients [28] and thus might have a predictive value in tumor progression of this disease. This is in line with reports on breast and ovarian cancer, where HLA-G or HLA-E expression correlated with a worse overall and event-free survival [15] and also in rectal and colon cancers, in which a combination of the immune-related markers HLA class I, HLA-G, HLA-E and FoxP3 reflect an immune escape mechanism [29, 30]. Using qPCR, flow cytometry and/or immunohistochemistry (IHC) this study analyzed the expression of HLA-E in RCC cell lines as well as in RCC lesions and correlated these data with clinical and immunologic parameters.

The results suggest that HLA-E might serve in combination with HLA-G as a prognostic biomarker and its status might contribute to a better selection of a subgroup of RCC patients with poor prognosis, who might benefit from individually tailored (immune) therapies.

## RESULTS

### Comparison of the mRNA expression of non-classical HLA class Ib molecules in RCC tumors with normal kidney epithelium

The expression of HLA-E, -F and -G transcripts in RCC tumors (T) and corresponding healthy normal kidney epithelium (N) was investigated by semi-quantitative PCR demonstrating higher expression levels of the non-classical HLA class Ib molecules in RCC tumorous tissues when compared to healthy control tissues.

**Figure 1:**
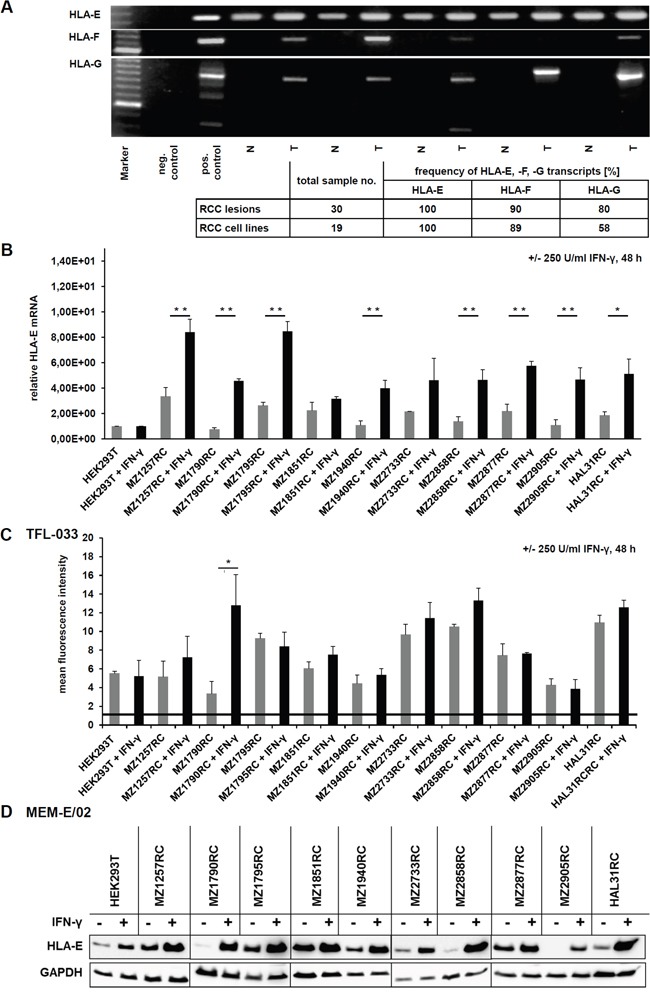
Characterization of HLA-E expression and IFN-γ inducibility in RCC lesions (T), corresponding healthy kidney epithelium (N) and RCC cell lines **A.** An exemplary picture of a agarose gel shows the determination of HLA-E, HLA-F and HLA-G mRNA in RCC tumors (T) and corresponding normal kidney epithelium (N) by semi-quantitative PCR, revealing, that HLA-G and HLA-F transcripts are only detectable in RCC tumors, while healthy normal kidney epithelium is lacking these transcripts. In contrast, HLA-E transcripts can be detected in all samples. However, the expression of HLA-E mRNA is lower in healthy control tissue when compared to the corresponding RCC lesions. The table below summarizes the expression frequency of HLA-E, HLA-F and HLA-G in RCC tumors and cell lines, which allows following ranking for detected frequency HLA-E > HLA-F > HLA-G. **B.** The HLA-E mRNA was quantified by qPCR in a panel of 10 selected RCC cell lines as well as in IFN- γ inert HEK293T cells without (grey) and with (black) IFN-γ stimulation for 48 h. HEK293T cells are reported for being insensitive for IFN-γ stimulation due to an IFN-γ receptor α-chain deficiency [31] and were therefore used for normalization. All of the investigated cell lines were positive for HLA-E transcript, and in 10 of 10 RCC cell lines an induction of HLA-E upon IFN-γ stimulation could be observed, which was statistical significant in 8 of 10 cases. The IFN-γ inert HEK293T did not respond to IFN-γ treatment. **C.** The induction of HLA-E protein upon IFN-γ stimulation for 48 h was analyzed in 10 RCC cell lines as well as in IFN-γ inert HEK293T cells [31] by intracellular flow cytometry by usage of the HLA-E-specific TFL-033 antibody, which only reacts with β_2_-m-free and peptide-free cytoplasmic HLA-E α-chain. In 6 of 10 RCC cell lines an induction of HLA-E protein could be detected, which was statistical significant in 1 of 10 cases. The HLA-E-specific antibody TFL-033 only recognizes unloaded HLA-E α-chain. The IFN-γ induced HLA-E protein that is already in complex of HLA-E α-chain, peptide and β_2_-m (within the cells and upon the cell surface) is not detected by this antibody. **D.** Western blot analyses of 10 RCC cell lines and the IFN-γ resistant HEK293T cells [31] +/− IFN-γ treatment for 48 h using the unspecific HLA-E antibody MEM-E/02, which also has a cross reactivity to some alleles of classical HLA class Ia molecules [24]. A strong induction of HLA-E protein upon IFN-γ stimulation was shown, which was actually much stronger without any IFN-γ mediated HLA-E mRNA induction observed by qPCR analysis and by flow cytometry using the anti-HLA-E specific antibody TFL-033. These data indicate that the MEM-E/02 antibody indeed shows cross reactivity to other also IFN-γ inducible HLA class I molecules. By usage of this MEM-E/02 antibody even the reported IFN-γ inert HEK293T cells show an enhanced signal upon IFN-γ signaling.

Whereas HLA-F and HLA-G were only detectable in RCC lesions, but not in normal kidney epithelium, the HLA-E expression was found in RCC lesions and to a weaker extent also in normal kidney epithelium. The frequency of the pathological HLA class Ib (HLA-E, -F and –G) transcription ranged from 80 % to 100 % in the 30 investigated RCC lesions and from 60 % to 100 % in the 19 analyzed RCC cell lines. Furthermore, the HLA-Ib mRNA levels can be ranked in RCC lesions (*in vivo*) and in established RCC cell lines (*in vitro*) as follows HLA-E > HLA-F > HLA-G (Figure 1A).

### IFN-γ inducibility of HLA-E expression in RCC cell lines

The constitutive and IFN-γ-induced expression of HLA-E mRNA and protein was determined in 10 in house us established primary RCC cell lines and in IFN-γ inert HEK293T cells. All RCC cell lines and HEK293T cells expressed HLA-E transcript, which was inducible upon IFN-γ treatment except HEK293T cells with known defects of the IFN-γ receptor α-chain [31]. The IFN-γ-mediated induction of HLA-E mRNA transcription was heterogeneous and statistical significant in 8 of 10 RCC cell lines (Figure 1B).

Due to the monospecificity of the mAb TFL-033 recognizing only β_2_-m and peptide-free intracellular heavy chains (HC) HLA-E monomers [24] this mAb was used to determine HLA-E expression in untreated and IFN-γ-treated RCC cell lines and as negative control in IFN-γ insensitive HEK293T cells. Despite flow cytometry in combination with the TFL-033 antibody neither detected a constitutive nor an IFN-γ-inducible expression of β_2_-m-free and peptide-free HLA-E α chains on the cell surface (data not shown), intracellular staining of constitutive and IFN-γ-inducible HLA-E α-chain (protein) was identified. A representative histogram is shown in Supplementary Figure 1. Corresponding to the results of HLA-E mRNA analyses (Figure 1B) HLA-E protein expression in HEK293T cells was not induced upon IFN-γ stimulation (Figure 1C).

At this point it is noteworthy that the IFN-γ induced HLA-E protein, which is already in complex with the peptide and β_2_-m within the cells or upon the cell surface is not detected by the TFL-033 antibody, which is actually a big benefit of the TFL-033 antibody, because also the peptide generation and loading by the antigen processing machinery (APM) components is enhanced by IFN-γ itself as well as the expression of many APM components and β_2_-m [32].

Western blot analyses of untreated and IFN-γ-treated HEK293T cells as well as of the 10 RCC cell lines using the anti-HLA-E antibody MEM-E/02 demonstrated a more pronounced HLA-E expression upon IFN-γ stimulation when compared to staining with the TFL-033 mAb. This could be explained by a cross reactivity of MEM-E/02 mAb with classical HLA class Ia molecules (Figure 1D) [24].

### The HLA-E specific mAb TFL-033 only reacts to peptide-free HLA-E α-chain

**Figure 2:**
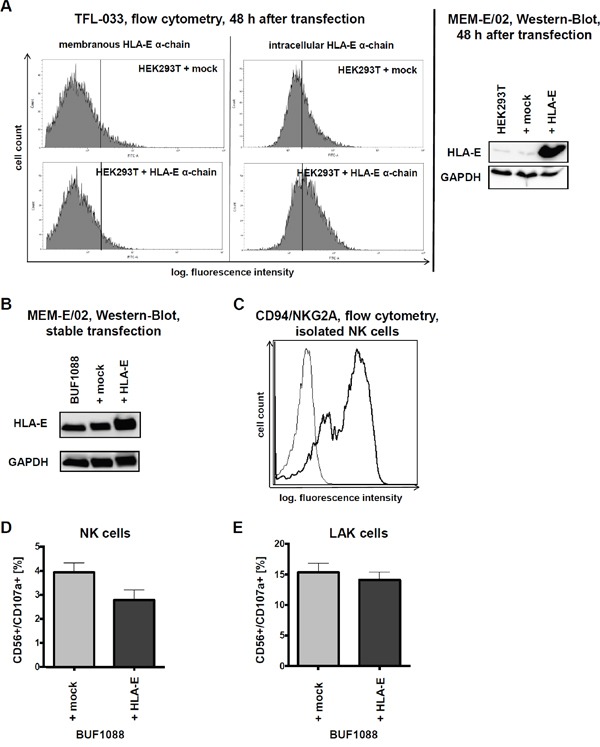
HLA-E overexpression and impact on immune recognition *in vitro* **A.** After 48 h of transient transfection of HEK293T cells with HLA-E expression vector and respective mock control an increased amount of HLA-E protein can be observed by intracellular flow cytometry with the TFL-033 antibody and by Western blotting with the MEM-E/02 antibody. Due to the direct comparison with the respective mock transfectants the results of both antibodies should regarded as sufficient specific, demonstrating furthermore the functionality of the HLA-E expression vector. **B.** Characterization of stable transfected BUF1088 cells (melanoma cell line) overexpressing HLA-E by Western-Blot (MEM-E/02 antibody). **C.** The presence of the main HLA-E receptor, the CD94/NKG2A heterodimer, on purified NK cells used for further *in vitro* cytotoxicity assays is demonstrated by flow cytometry. **D.** The CD107a degranulation assay shows a HLA-E-dependent lysis of BUF1088 transfectants by purified NK cells, expressed as mean of three experiments with NK cells of three different donors. The expressed results are not statistically significant. **E.** In analogy to Figure 2D the result of a CD107a degranulation assay with LAK cells demonstrates, that the inhibitory effects of HLA-E on immune effector cells can be abolished by high doses of IL-2 (*in vitro*). The expressed results are not statistically significant.

Membranous and intracellular HLA-E expression was also determined after transient transfection of a HLA-E expression vector into HEK293T cells using flow cytometry. Only an intracellular HLA-E-specific staining was detected with the TFL-033 antibody (Figure 1B, 1C and Figure 2A). The functionality of the HLA-E expression vector was proven by its transient transfection into HEK293T cells followed by Western blotting analyses (MEM-E/02) in comparison to untransfected and mock vector-transfected HEK293T cells (Figure 2A). Due to these controls the cross reactivity of MEM-E/02 can be neglected.

### Stable HLA-E transfectants and *in vitro* cytotoxicity assays

Recently, it has been shown that HLA-E presented peptides influence the affinity of HLA-E for the different activating or inhibitory HLA-E receptors on immune effector cells [25].

The immune modulatory functions of HLA-E were determined in the stable transfected HLA-E overexpressing (HLA-G negative) melanoma cell line BUF1088 (Figure 2B). Therefore HLA-E expressing BUF1088 and controls were co-cultured for 4 h with NK and LAK cells, before cytotoxicity was determined using the CD107a degranulation assay. As shown in Figure 2C, the main inhibitory HLA-E receptor CD94/NKG2A is expressed on the applied NK cells.

As expected HLA-E overexpression caused a reduced CD107a degranulation of NK cells (Figure 2D). Despite LAK cells showed an enhanced lysis capability, the cytotoxicity was not reduced in the presence of HLA-E-overexpressing tumor cells (Figure 2E). The increased effector potency of LAK cultures did overcome the inhibitory activity by the CD94/NKG2A engagement, but with modest and reproducible effects. Therefore HLA-E overexpression in cancer *in vivo* might provide a potential tumor immune escape mechanism due to possible long term effects.

### Determination of the HLA-E expression in RCC tumors

**Figure 3:**
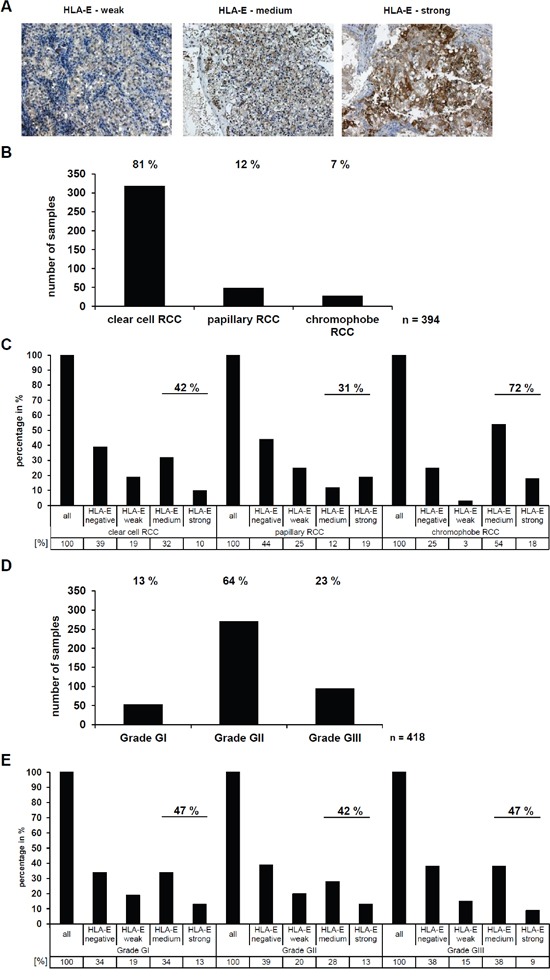
Determination and correlation of the HLA-E expression in RCC tumors (*in vivo*) with clinical parameters **A.** The HLA-E-specific TFL-033 antibody was used for immunohistochemical staining of >450 RCC tumors spotted on a tissue microarray (TMA). Exemplary three HLA-E positive RCC tumors are shown with a differential HLA-E expression ranging from weak, medium and strong. The staining with the TFL-033 antibody allows statements about the HLA-E expression without any distortion by a potential cross reactivity to other HLA class I molecules [24], which even could have *per se* a higher abundance or by a differential expression of APM components like TAP1, TAP2, TPN or B2M, which are often down regulated in tumor diseases [3]. Therefore, the TFL-033 staining is ideal for investigation of the HLA-E expression, only. **B.** The applied TMA consists of RCC tumor samples of the different RCC subtypes. For further analyses the three major subtypes clear cell RCC, papillary RCC and chromophobe RCC were used. The statistical distribution of these RCC subtypes was analyzed and can be ranked as follows: clear cell (81 %) > papillary (12 %) > chromophobe (7%). Other RCC subtypes or unspecified RCC tumors were excluded for further analyses, therefore the absolute number of analyzed RCC samples is n = 394. **C.** The HLA-E expression in the three different RCC subtypes (n = 394; Figure 3B) was analyzed by IHC shows a strong correlation between HLA-E expression and the chromophobe RCC subtype, when compared to clear cell and papillary RCCs. **D.** The RCC tumors were further analyzed for their tumor grading according to the 2004 World Health Organization (WHO) classification (Eble JN, Sauter G, Epstein JI, Sesterhenn IA. Pathology and Genetics of Tumours of the Urinary System and Male Genital Organs). The statistical distribution can be ranked as follows: WHO Grade GII (64%) > WHO Grade GIII (23%) > WHO Grade GI (13%). Unspecified RCC samples were excluded, therefore the absolute number of analyzed RCC samples is n = 418. **E.** No correlation between the HLA-E expression and the WHO grade (n = 418; Figure 3D) of the analyzed RCC tumors could be observed.

The HLA-E expression of RCC tumors was also analyzed on a RCC tissue microarray (TMA) with >450 RCC samples applying immunohistochemistry (IHC) by staining the intracellular HLA-E α-chains by usage of the TFL-033 mAb. Patients and tumor characteristics of this TMA have been recently published [27]. Representative staining of RCC lesions with a different HLA-E expression pattern are shown in Figure 3A. In all HLA-E positive samples only a cytoplasmic (peptide free HLA-E α-chain), but not a membranous staining pattern of HLA-E could be detected ranging from low (+), medium (++) to high (+++) HLA-E expression.

Interestingly, the HLA-E expression strongly varied between the different RCC subtypes (Figure 3B), since 31 % of papillary RCC, 42 % of clear cell RCC, and 72 % of chromophobe RCC expressed medium to strong HLA-E levels (Figure 3C).

Next to this it was investigated whether a correlation between HLA-E expression in RCC lesions and WHO tumor grading exists, which was statistically significant for HLA-G as recently reported [27]. About 13 % of the RCC lesions were grade GI, 64 % grade GII and 23 % grade GIII (Figure 3D). No correlation between the HLA-E expression and the WHO tumor grading could be observed (Figure 3E).

### Correlation of HLA-E expression with immune cell infiltration

**Figure 4:**
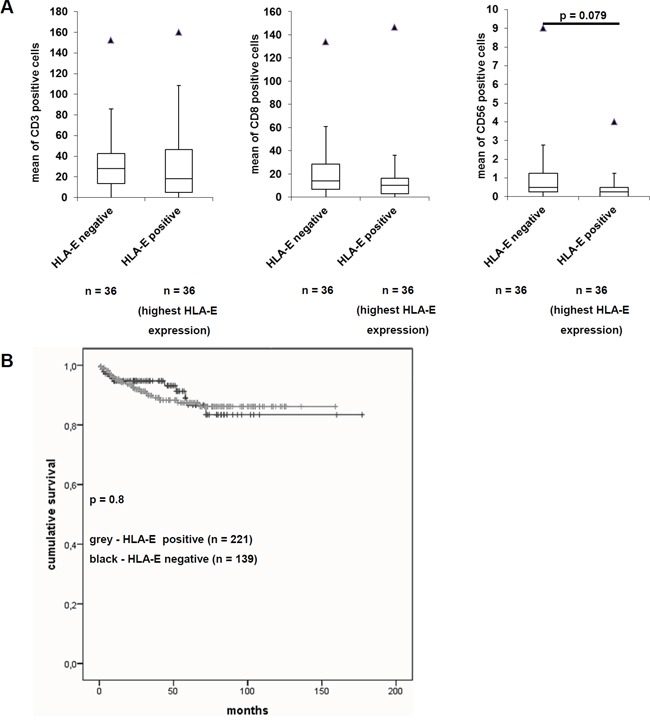
Effects of HLA-E expression on the immunoscore and on the disease specific survival of RCC patients **A.** Immunohistochemical staining of the immune cell markers CD3, CD8 and CD56 were performed on the TMA and correlated to the HLA-E expression as recently reported by Jasinski-Bergner et al., 2015 [27]. The immune cell markers CD4, CD25, CD69 and FoxP3 were only barely detectable (data not shown). While the immune cell markers CD3 and CD8 showed no difference in HLA-E negative and HLA-E positive tumors, the immune cell marker CD56 was decreased in HLA-E positive tumors. The fact that only CD56 and not CD3 show an inverse correlation to HLA-E expression indicates NK cells and excludes NKT cells. However, the down regulation of NK cells in HLA-E positive tumors was almost significant (p = 0.079). **B.** The Kaplan-Meier-Plot for RCC patients reveals that HLA-E expression is not correlated with a worse disease specific survival (p = 0.8).

In order to determine an association of immune cell infiltration with HLA-E expression, the TMA was further stained with mAbs specific for T cell subsets and NK cells as well as for activation markers of these immune cells. Thus, 36 HLA-E-negative and 36 HLA-E-positive RCC lesions were analyzed for their immune cell infiltration, in particular for CD3^+^, CD8^+^ and CD56^+^ immune effector cells. The data are presented as Box-Whiskers-Plots (Figure 4A). A statistically significant correlation between HLA-G expression with tumor infiltrating CD3 positive and CD8 positive cells, but no difference for CD56 positive cells was recently reported by Jasinski-Bergner and co-authors [27]. In contrast, HLA-E expression could not be correlated to the immune cell markers CD3 and CD8, but an almost significant (p = 0.079) correlation with decreased CD56^+^ cell infiltration was shown. Moreover a correlation with NKT cells could be excluded, favoring an inverse correlation of HLA-E expression and infiltrating NK cells in RCC. Other markers like CD4, FOXP3, CD69 and CD25 showed no HLA-E dependency.

### Association between HLA-E expression and disease-specific survival of RCC patients

Due to the availability of clinicopathologic parameters, HLA-E expression was analyzed for patients’ disease-specific survival. As shown in Figure 4B, HLA-E expression did not lead to a decreased disease-specific survival of RCC patients when compared to the HLA-E negative cohort. The effect of HLA-E expression to a decreased disease specific survival of RCC patients was not significant (p = 0.8).

Next the impact of concordant HLA-E and HLA-G protein expression on RCC cells leading to two independent mechanisms of immune evasion was determined. Although there exists no correlation between HLA-E expression with membranous or cytoplasmic HLA-G expression, 27 % of RCC lesions were HLA-E and HLA-G double positive.

HLA-G interacts with ILT2, ILT4 and KIR2DL4 and HLA-E binds to CD94/NKG2A, -B and –C. However, double positive tumors (HLA-G and HLA-E) could *per se* show additive effects of both immune-modulatory molecules to increase the chance of immune evasion indicating that both targets (HLA-G and HLA-E) should be discussed as potential targets for immune therapies in RCC patients (Supplementary Figure 2).

## DISCUSSION

Different immune escape mechanisms have been identified in RCC thereby leading to a reduced recognition of tumor cells by immune effector cells, which is associated with a poor prognosis of these patients. These include a high frequency of the expression of immunomodulatory HLA-G and HLA-E [26–28, 33], a downregulation of HLA class Ia expression due to altered APM component expression and/or a deficient IFN-γ signaling of RCC cells [3, 34–36]. Furthermore, tumors with loss of HLA class Ia expression often express HLA-G and/or HLA-E thereby evading both CTL and NK cell recognition. Despite a high frequency of T cell infiltration significantly correlated to HLA-G expression a reduced patients’ survival was found [27, 37, 38]. Regulatory FoxP3^+^ T cells (Treg) have immune suppressive activity by inhibiting host anti-tumor responses. This was often associated with an unfavorable prognosis of a wide range of human cancers, including breast, lung and colon carcinoma as well as RCC [39, 40].

Our study monitored for the first time a cohort of 453 RCC lesions for HLA-E expression using the HLA-E-specific TFL-033 mAb. While other anti-HLA-E antibodies have been reported to be cross-reactive to alleles of other HLA class I molecules [24], the TFL-033 recognizes an unique epitope in the peptide binding cleft of β_2_-m-free and peptide-free HLA-E α-chain.

It is noteworthy that membranous HLA class I expression is a result of peptide processing and loading (e.g. TAP1, TAP2. TPN) followed by vesicular transport to the cell surface and is additionally β_2_-m dependent. These factors are often downregulated in certain tumor entities [3] and might influence the detection of HLA-E as complex with peptide and β_2_-m on the cell surface.

Furthermore, the genotype of the HLA-E allele and the amino acid sequence of the presented peptide contribute to the assembly of the trimeric complex of HLA-E α-chain, processed and loaded peptide and β_2_-m [41, 42].

In our study HLA-E mRNA and protein expression frequently occurred in RCC cell lines and tumors and could be correlated in particular to the chromophobe RCC subtype. There exists no correlation between HLA-E expression with the WHO tumor grading, HLA-G expression or an altered T cell infiltration. In contrast, HLA-E expression was almost significant associated with an inverse infiltration of NK cells, but not linked to a decreased disease-specific survival of RCC patients. However, analysis of the effect of HLA-E expression on the overall survival should be addressed in further studies. It is noteworthy, that these data are in accordance with a recent paper by Andersson and co-authors on ovarian carcinoma [11].

Thus it could be postulated that RCC lesions might progress due to the lack of immune surveillance by both NK and T cells (HLA-G, HLA-E). About 27 % of RCC lesions on the TMA concordantly expressed HLA-G and –E, which might display an even worse clinical outcome due to potential additive effects. While HLA-G binds the inhibitory receptors ILT2, ILT4 and KIR2DL4, HLA-E binds the heterodimer of CD94/NKG2A, -B and -C [4, 43]. Furthermore, the function of HLA-E on tumor cells was assessed focusing on the effect of HLA-E on NK and LAK cell cytotoxicity. HLA-E overexpression has been shown to negatively interfere with innate immune responses thereby protecting cells from susceptibility to lysis, which resulted in resistance of HLA-E^+^ tumor cells to NK cell-mediated cytotoxicity [20].

Currently, immunotherapies for RCC patients include antibodies directed against checkpoint inhibitors, e.g. CTLA-4, PD-1 or PD-L1, the application of bispecific antibodies or chimeric antigen receptors in combination with NK or T cell transfer into the patient [44–46]. However, these therapies should address the question if the targeted tumor cells express immune modulatory molecules that weaken or even completely prevent the immune response.

A combination of HLA-G and HLA-E as markers might lead to a better prognostic value than the analysis of a single immune marker. Indeed, various studies demonstrated that a single immune cell marker is not sufficient for selection of high risk patients or treatment allocation [47, 48]. Furthermore, it is noteworthy that only a snap shot of the ongoing process of cancer immune editing could be demonstrated in the primary RCC lesions at the time of resection. Due to the specific HLA-E antibody used, RCC lesions have been shown for the first time to express intact HLA-E antigens. Thus, this study circumvents limitations of previous reports applying HLA-E antibodies also recognizing HC of other HLA class I antigens.

## MATERIALS AND METHODS

### Cell lines, cell culture and IFN-γ stimulation

The human embryonal kidney cell line HEK293T (ATCC® CRL-3216™) was purchased from the American Type Culture Collection (ATCC, Manassas, USA), whereas the ten RCC cell lines analyzed were established in the institute and have been published elsewhere [26, 49, 50]. All RCC cell lines were cultured in Dulbecco's modified Eagles medium (DMEM, Life Technologies, Grand Island, NY, USA) supplemented with 10 % fetal bovine serum, 2 mM L-glutamine (Lonza, Basel, Switzerland), 1 % penicillin/streptomycin (V/V; PAA, Pasching, Austria), 25 mM HEPES (c-c-pro, Oberdorla, Germany).

For IFN-γ (PAN-Biotech, Aidenbach, Germany) stimulation RCC cells were treated with 250 U IFN-γ/ml for 48 h. After 24 h the medium was replaced by new medium supplemented with 250 U/ml IFN-γ.

### RNA extraction, cDNA synthesis and qPCR

Total RNA was extracted from cell pellets employing TRIzol Reagent (Life Technologies), digested with DNase I (NEB, Ipswich, MA, USA) and applied for cDNA synthesis utilizing the RevertAidTM H Minus First Strand cDNA synthesis kit (Life Technologies) as recently reported in Jasinski-Bergner and co-authors [27].

HLA-E transcript was detected using the HLA-E-specific primers E.312 (forward: 5′-TGCGCGGCTACTACAATCAG-3′) and E.1052 (reverse: 5′-TGTCGCTCCACTCAGCCTTC-3′). The HLA-E transcript was normalized to GAPDH (forward 5′- CAAGGTCATCCATGACAACTTTG-3′ and reverse 5′- GTCCACCACCCTGTTGCTGTAG-3′ by Thermo Scientific). The qPCR reactions were performed in a Rotorgene cycler (Qiagen, Hilden, Germany) at 95°C for 5 min followed by 40 cycles of 95°C for 30 s, 60°C for 30 s and 72°C for 40 s.

HLA-E, HLA-F and HLA-G mRNA detection from biopsies were further characterized by semi-quantitative PCR [49] using the Titan One Tube RT-PCR System (Roche Diagnostics, Heidelberg, Germany) and specific primers for HLA-G: G.257 (forward: 5′-GGAAGAGGAGACACGGAACA-3′) and G.1225 (reverse: 5′-TGAGACAGAGACGGAGACAT-3′) by Real et al., 1999 [51], T_m_ 61°C and resulting PCR products were size fractionated on 1% ethidium bromide containing agarose gels [49]. For the detection of HLA-F the following PCR primers were applied: forward 5′-CAGTTCCCAGGTTCTAAAGTCC-3′ and reverse 5′-AACGTGTGCCTTTGGAGGA-3′.

### Cloning and transfection of HLA-E expression vector

The coding sequence of HLA-E was amplified by PCR applying the Q5^®^ High-Fidelity DNA Polymerase (NEB, Ipswich, MA, USA) using the forward primer 5′-AAAGAATTCAATCAGCGTCGCCACGACTCC-3′ and reverse primer 5′-AAACTCGAGAGGCAGCTGTGCATCTCAGTC-3′. The resulting PCR product was then cloned via EcoRI and XhoI (NEB) into the pCMV-IRES-neo^R^ expression vector as previously described [52]. HEK293T cells were transiently, BUF1088 melanoma cells stably transfected with the HLA-E expression vector or the respective mock vector, employing the Effectene transfection reagent (Qiagen, Hilden, Germany). Geneticin-resistant melanoma cells were selected [53].

### Flow cytometry

For flow cytometric analyses of the HLA-E surface expression after transient or stable overexpression of HLA-E α-chain the mAbs anti-HLA-E MEM-E/02 (EXBIO, Prague, Czech Republic) and TFL-033 were applied. Briefly, cells were harvested, washed with PBS and incubated with the respective mAb for 30 min at room temperature, washed with PBS followed by a second incubation for 30 min at room temperature using an AlexaFluor 488-labelled mAb as secondary antibody. After an additional washing step the stained cells were analyzed on a flow cytometer (LSRFortessa, BD, Franklin Lakes, NJ, USA). The data were presented as mean specific fluorescence intensity (MFI) or as histograms using the Kaluza Analysis Software (Beckman-Coulter, Brea, CA, USA).

For the detection of intracellular HLA-E the cells were fixed for 30 min at room temperature with 4 % paraformaldehyde, washed and subsequently treated with permeabilizing buffer (methanol, -20°C) for 30 min at 4°C prior to antibody staining [50].

### Protein extraction and western blot analysis

Proteins were extracted from frozen cell pellets and Western blot analyses using 50 μg of total protein/lane was performed as recently described [54] using the anti-HLA-E antibody MEM-E/02 (Exbio; Prague, Czech Republic) and TFL-033, respectively. The anti-GAPDH mAb 14C10 (Cell Signaling, Danvers, MA, USA) served as loading control. For detection, HRP-conjugated secondary antibodies (DAKO, Hamburg, Germany) and the LumiLight Western blotting substrate (Roche Diagnostics, Basel, Switzerland) were used. The staining was recorded with a CCD camera system (Fuji BAS 3000, Fuji, Valhalla, NY, USA).

### CD107a degranulation assay

CD56^+^ NK cells were isolated from PBMCs of healthy blood donors after Ficoll gradient centrifugation using the NK cell isolation kit (Miltenyi Biotec, Bergisch Gladbach, Germany). The purity of cells was tested >95 %. NK cells as well as overnight high dose IL-2 (2000 U/ml, Merck Millipore, Darmstadt, Germany)-treated lymphokine-activated killer (LAK) cells were cultured in X-VIVO15 (Life Technologies) supplemented with 2 mM L-glutamine and 1 % penicillin/streptomycin.

For the CD107a degranulation assay effector (E) and target (T) cells were co-cultured for 4 h at 37°C in an E:T ratio of 20:1 followed by the analysis of the presence CD107a on the cell surface of CD56^+^ cells. NK cells were additionally tested for the presence of CD94 on the cell surface. Flow cytometric analysis was performed using the BD FACSAria Fusion (BD) and the FlowJo Software.

### Tissue microarrays (TMA) and tissue collection and immunohistochemistry (IHC)

The generation of the tissue microarrays (TMA) has been recently described [55]. Details of the TMA consisting of 453 formalin-fixed, paraffin-embedded RCC tissues along with the corresponding normal kidney tissues not directly adjacent to the tumor as well as the patients’ and tumor characteristics have been described [27]. The study was approved by the Ethical committee of the University of Erlangen-Nuremberg (Germany) and conducted to the principles expressed in the declaration of Helsinki.

Immunohistochemical staining of the TMA for HLA-E performed on 5 μM sections using the mAb TFL-033 [24]. The tissue sections were incubated for 60 min at room temperature followed by a staining with a goat anti-mouse lgG as secondary mAb for 30 min at room temperature. Then the sections were incubated with immunoperoxidase, washed and developed with diaminobenzidine (DAB Kit, Vector). Slides were counterstained with hematoxylin. Negative controls were obtained by omitting the primary antibody. Microscopic analysis of the staining was performed by two independent observers including one pathologist specialized on uro-pathology, who scored the intensity of HLA-E staining as absent, weak, moderate and strong. Staining for HLA-G and immune cell infiltrates has been recently described [27].

### Statistical analysis

Statistical analyses were performed using the program SPSS (version 17.0 for Windows). The student's t-test and the chi square-test were used to evaluate the associations between HLA-E and HLA-G expression and tumor immune cell infiltration.

For survival analyses, log-rank test was applied using IBM SPSS Statistics 21. Survival probabilities were plotted following the Kaplan-Meier method.

## SUPPLEMENTARY MATERIAL FIGURES



## Abbreviations

APC: antigen presenting cell
APM: antigen processing machinery
β2-m: β2-microglobuline
CTL: cytotoxic T lymphocyte
HC: heavy chain
HLA: human leukocyte antigen
FITC: fluorescein isothiocyanate
IFN: interferon
IHC: immunohistochemistry
ILT: immunoglobulin-like transcript
KIR: killer cell immunoglobulin-like receptors
LAK: lymphokine-activated killer
mAb: monoclonal antibody
MFI: mean fluorescence intensity
miR: microRNA
NK: natural killer
NKR-L: NK cell receptor ligand
OS: overall survival
PBL: peripheral blood lymphocyte
PBMC: peripheral blood mononuclear cells
PCR: polymerase chain reaction
RCC: renal cell carcinoma
sHLA-G: soluble HLA-G
SNP: single nucleotide polymorphism
TAP: transporter associated with antigen processing
TCR: T cell receptor
TIL: tumor-infiltrating lymphocyte
TLR: toll-like receptor
TMA: tissue microarray
Treg: regulatory T cell
UTR: untranslated region.

## References

[1] Cavallo F, De Giovanni C, Nanni P, Forni G, Lollini PL (2011). 2011: the immune hallmarks of cancer. Cancer Immunol Immunother.

[2] Gong F, Song S, Lv G, Pan Y, Zhang D, Jiang H (2012). Human leukocyte antigen E in human cytomegalovirus infection: friend or foe?. Acta Biochim Biophys Sin (Shanghai).

[3] Bukur J, Jasinski S, Seliger B (2012). The role of classical and non-classical HLA class I antigens in human tumors. Semin Cancer Biol.

[4] Braud VM, Allan DS, O'Callaghan CA, Soderstrom K, D'Andrea A, Ogg GS, Lazetic S, Young NT, Bell JI, Phillips JH, Lanier LL, McMichael AJ (1998). HLA-E binds to natural killer cell receptors CD94/NKG2A, B and C. Nature.

[5] Speiser DE, Valmori D, Rimoldi D, Pittet MJ, Lienard D, Cerundolo V, MacDonald HR, Cerottini JC, Romero P (1999). CD28-negative cytolytic effector T cells frequently express NK receptors and are present at variable proportions in circulating lymphocytes from healthy donors and melanoma patients. European journal of immunology.

[6] Braud VM, Aldemir H, Breart B, Ferlin WG (2003). Expression of CD94-NKG2A inhibitory receptor is restricted to a subset of CD8+ T cells. Trends Immunol.

[7] Derre L, Corvaisier M, Charreau B, Moreau A, Godefroy E, Moreau-Aubry A, Jotereau F, Gervois N (2006). Expression and release of HLA-E by melanoma cells and melanocytes: potential impact on the response of cytotoxic effector cells. J Immunol.

[8] Rizzo R, Trentini A, Bortolotti D, Manfrinato MC, Rotola A, Castellazzi M, Melchiorri L, Di Luca D, Dallocchio F, Fainardi E, Bellini T (2013). Matrix metalloproteinase-2 (MMP-2) generates soluble HLA-G1 by cell surface proteolytic shedding. Mol Cell Biochem.

[9] Borrego F, Ulbrecht M, Weiss EH, Coligan JE, Brooks AG (1998). Recognition of human histocompatibility leukocyte antigen (HLA)-E complexed with HLA class I signal sequence-derived peptides by CD94/NKG2 confers protection from natural killer cell-mediated lysis. Journal of Experimental Medicine.

[10] Lee N, Goodlett DR, Ishitani A, Marquardt H, Geraghty DE (1998). HLA-E surface expression depends on binding of TAP-dependent peptides derived from certain HLA class I signal sequences. J Immunol.

[11] Andersson E, Poschke I, Villabona L, Carlson JW, Lundqvist A, Kiessling R, Seliger B, Masucci GV (2016). Non-classical HLA-class I expression in serous ovarian carcinoma: Correlation with the HLA-genotype, tumor infiltrating immune cells and prognosis. Oncoimmunology.

[12] Palmisano GL, Contardi E, Morabito A, Gargaglione V, Ferrara GB, Pistillo MP (2005). HLA-E surface expression is independent of the availability of HLA class I signal sequence-derived peptides in human tumor cell lines. Hum Immunol.

[13] Teklemariam T, Zhao L, Hantash BM (2012). Full-length HLA-G1 and truncated HLA-G3 differentially increase HLA-E surface localization. Hum Immunol.

[14] Silva TG, Crispim JC, Miranda FA, Hassumi MK, de Mello JM, Simoes RT, Souto F, Soares EG, Donadi EA, Soares CP (2011). Expression of the nonclassical HLA-G, HLA-E molecules in laryngeal lesions as biomarkers of tumor invasiveness. Histol Histopathol.

[15] de Kruijf EM, Sajet A, van Nes JG, Natanov R, Putter H, Smit VT, Liefers GJ, van den Elsen PJ, van de Velde CJ, Kuppen PJ (2010). HLA-E and HLA-G expression in classical HLA class I-negative tumors is of prognostic value for clinical outcome of early breast cancer patients. J Immunol.

[16] Talebian Yazdi M, van Riet S, van Schadewijk A, Fiocco M, van Hall T, Taube C, Hiemstra PS, van der Burg SH (2016). The positive prognostic effect of stromal CD8+ tumor-infiltrating T cells is restrained by the expression of HLA-E in non-small cell lung carcinoma. Oncotarget.

[17] Hiraoka N, Onozato K, Kosuge T, Hirohashi S (2006). Prevalence of FOXP3+ regulatory T cells increases during the progression of pancreatic ductal adenocarcinoma and its premalignant lesions. Clin Cancer Res.

[18] Guo ZY, Lv YG, Wang L, Shi SJ, Yang F, Zheng GX, Wen WH, Yang AG (2015). Predictive value of HLA-G and HLA-E in the prognosis of colorectal cancer patients. Cell Immunol.

[19] Zhen ZJ, Ling JY, Cai Y, Luo WB, He YJ (2013). Impact of HLA-E gene polymorphism on HLA-E expression in tumor cells and prognosis in patients with stage III colorectal cancer. Med Oncol.

[20] Lo Monaco E, Tremante E, Cerboni C, Melucci E, Sibilio L, Zingoni A, Nicotra MR, Natali PG, Giacomini P (2011). Human leukocyte antigen E contributes to protect tumor cells from lysis by natural killer cells. Neoplasia.

[21] Nguyen S, Beziat V, Dhedin N, Kuentz M, Vernant JP, Debre P, Vieillard V (2009). HLA-E upregulation on IFN-gamma-activated AML blasts impairs CD94/NKG2A-dependent NK cytolysis after haplo-mismatched hematopoietic SCT. Bone Marrow Transplant.

[22] Tremante E, Lo Monaco E, Ingegnere T, Sampaoli C, Fraioli R, Giacomini P (2015). Monoclonal antibodies to HLA-E bind epitopes carried by unfolded beta2 m-free heavy chains. European journal of immunology.

[23] Jucaud V, Ravindranath MH, Terasaki PI (2015). Immunobiology of HLA Class-Ib Molecules in Transplantation. SOJ Immunol.

[24] Sasaki T, Ravindranath MH, Terasaki PI, Freitas MC, Kawakita S, Jucaud V (2014). Gastric cancer progression may involve a shift in HLA-E profile from an intact heterodimer to beta2-microglobulin-free monomer. Int J Cancer.

[25] Ravindranath MH, Terasaki PI, Pham T, Jucaud V (2015). The Monospecificity of Novel Anti-HLA-E Monoclonal Antibodies Enables Reliable Immunodiagnosis, Immunomodulation of HLA-E, and Upregulation of CD8+ T Lymphocytes. Monoclon Antib Immunodiagn Immunother.

[26] Dunker K, Schlaf G, Bukur J, Altermann WW, Handke D, Seliger B (2008). Expression and regulation of non-classical HLA-G in renal cell carcinoma. Tissue antigens.

[27] Jasinski-Bergner S, Stoehr C, Bukur J, Massa C, Braun J, Huttelmaier S, Spath V, Wartenberg R, Legal W, Taubert H, Wach S, Wullich B, Hartmann A, Seliger B (2015). Clinical relevance of miR-mediated HLA-G regulation and the associated immune cell infiltration in renal cell carcinoma. Oncoimmunology.

[28] Kren L, Valkovsky I, Dolezel J, Capak I, Pacik D, Poprach A, Lakomy R, Redova M, Fabian P, Krenova Z, Slaby O (2012). HLA-G and HLA-E specific mRNAs connote opposite prognostic significance in renal cell carcinoma. Diagn Pathol.

[29] Reimers MS, Engels CC, Putter H, Morreau H, Liefers GJ, van de Velde CJ, Kuppen PJ (2014). Prognostic value of HLA class I, HLA-E, HLA-G and Tregs in rectal cancer: a retrospective cohort study. BMC Cancer.

[30] Zeestraten EC, Reimers MS, Saadatmand S, Goossens-Beumer IJ, Dekker JW, Liefers GJ, van den Elsen PJ, van de Velde CJ, Kuppen PJ (2014). Combined analysis of HLA class I, HLA-E and HLA-G predicts prognosis in colon cancer patients. Br J Cancer.

[31] Kong XF, Vogt G, Chapgier A, Lamaze C, Bustamante J, Prando C, Fortin A, Puel A, Feinberg J, Zhang XX, Gonnord P, Pihkala-Saarinen UM, Arola M, Moilanen P, Abel L, Korppi M (2010). A novel form of cell type-specific partial IFN-gammaR1 deficiency caused by a germ line mutation of the IFNGR1 initiation codon. Hum Mol Genet.

[32] Seliger B, Dunn T, Schwenzer A, Casper J, Huber C, Schmoll HJ (1997). Analysis of the MHC class I antigen presentation machinery in human embryonal carcinomas: evidence for deficiencies in TAP, LMP and MHC class I expression and their upregulation by IFN-gamma. Scandinavian journal of immunology.

[33] Li BL, Lin A, Zhang XJ, Zhang X, Zhang JG, Wang Q, Zhou WJ, Chen HX, Wang TJ, Yan WH (2009). Characterization of HLA-G expression in renal cell carcinoma. Tissue antigens.

[34] Atkins D, Ferrone S, Schmahl GE, Storkel S, Seliger B (2004). Down-regulation of HLA class I antigen processing molecules: an immune escape mechanism of renal cell carcinoma?. J Urol.

[35] Dovhey SE, Ghosh NS, Wright KL (2000). Loss of interferon-gamma inducibility of TAP1 and LMP2 in a renal cell carcinoma cell line. Cancer Res.

[36] Zhang Q, Zhang L, Li L, Wang Z, Ying J, Fan Y, Xu B, Wang L, Liu Q, Chen G, Tao Q, Jin J (2014). Interferon regulatory factor 8 functions as a tumor suppressor in renal cell carcinoma and its promoter methylation is associated with patient poor prognosis. Cancer Lett.

[37] Geissler K, Fornara P, Lautenschlager C, Holzhausen HJ, Seliger B, Riemann D (2015). Immune signature of tumor infiltrating immune cells in renal cancer. Oncoimmunology.

[38] Hase S, Weinitschke K, Fischer K, Fornara P, Hoda R, Unverzagt S, Seliger B, Riemann D (2011). Monitoring peri-operative immune suppression in renal cancer patients. Oncol Rep.

[39] Lindau D, Gielen P, Kroesen M, Wesseling P, Adema GJ (2013). The immunosuppressive tumour network: myeloid-derived suppressor cells, regulatory T cells and natural killer T cells. Immunology.

[40] Whiteside TL (2015). Clinical Impact of Regulatory T cells (Treg) in Cancer and HIV. Cancer Microenviron.

[41] Celik AA, Kraemer T, Huyton T, Blasczyk R, Bade-Doding C (2016). The diversity of the HLA-E-restricted peptide repertoire explains the immunological impact of the Arg107Gly mismatch. Immunogenetics.

[42] Lauterbach N, Wieten L, Popeijus HE, Vanderlocht J, van Zon PM, Voorter CE, Tilanus MG (2015). Peptide-induced HLA-E expression in human PBMCs is dependent on peptide sequence and the HLA-E genotype. Tissue antigens.

[43] Carosella ED, Gregori S, LeMaoult J (2011). The tolerogenic interplay(s) among HLA-G, myeloid APCs, and regulatory cells. Blood.

[44] Andersen R, Donia M, Westergaard MC, Pedersen M, Hansen M, Svane IM (2015). Tumor infiltrating lymphocyte therapy for ovarian cancer and renal cell carcinoma. Hum Vaccin Immunother.

[45] Cohen J, Sznol M (2015). Therapeutic combinations of immune-modulating antibodies in melanoma and beyond. Semin Oncol.

[46] Combe P, de Guillebon E, Thibault C, Granier C, Tartour E, Oudard S (2015). Trial Watch: Therapeutic vaccines in metastatic renal cell carcinoma. Oncoimmunology.

[47] Menon AG, Morreau H, Tollenaar RA, Alphenaar E, Van Puijenbroek M, Putter H, Janssen-Van Rhijn CM, Van De Velde CJ, Fleuren GJ, Kuppen PJ (2002). Down-regulation of HLA-A expression correlates with a better prognosis in colorectal cancer patients. Lab Invest.

[48] Watson NF, Ramage JM, Madjd Z, Spendlove I, Ellis IO, Scholefield JH, Durrant LG (2006). Immunosurveillance is active in colorectal cancer as downregulation but not complete loss of MHC class I expression correlates with a poor prognosis. Int J Cancer.

[49] Bukur J, Rebmann V, Grosse-Wilde H, Luboldt H, Ruebben H, Drexler I, Sutter G, Huber C, Seliger B (2003). Functional role of human leukocyte antigen-G up-regulation in renal cell carcinoma. Cancer Res.

[50] Quandt D, Jasinski-Bergner S, Muller U, Schulze B, Seliger B (2014). Synergistic effects of IL-4 and TNFalpha on the induction of B7-H1 in renal cell carcinoma cells inhibiting allogeneic T cell proliferation. J Transl Med.

[51] Real LM, Cabrera T, Collado A, Jimenez P, Garcia A, Ruiz-Cabello F, Garrido F (1999). Expression of HLA G in human tumors is not a frequent event. Int J Cancer.

[52] Kallfelz M, Jung D, Hilmes C, Knuth A, Jaeger E, Huber C, Seliger B (1999). Induction of immunogenicity of a human renal-cell carcinoma cell line by TAP1-gene transfer. Int J Cancer.

[53] Respa A, Bukur J, Ferrone S, Pawelec G, Zhao Y, Wang E, Marincola FM, Seliger B (2011). Association of IFN-gamma signal transduction defects with impaired HLA class I antigen processing in melanoma cell lines. Clin Cancer Res.

[54] Jasinski-Bergner S, Stehle F, Gonschorek E, Kalich J, Schulz K, Huettelmaier S, Braun J, Seliger B (2014 (b)). Identification of 14-3-3beta gene as a novel miR-152 target using a proteome-based approach. J Biol Chem.

[55] Kononen J, Bubendorf L, Kallioniemi A, Barlund M, Schraml P, Leighton S, Torhorst J, Mihatsch MJ, Sauter G, Kallioniemi OP (1998). Tissue microarrays for high-throughput molecular profiling of tumor specimens. Nat Med.

