# Supplementary material for: HLA-E expression and its clinical relevance in human renal cell carcinoma
Source: Oncotarget. 2016 Aug 31;7(41):67360–72. doi: 10.18632/oncotarget.11744 (PMC5341881; doi:10.18632/oncotarget.11744)
Supplement: Supplementary file 1 [file oncotarget-07-67360-s001.pdf]

# HLA-E expression and its clinical relevance in human renal cell carcinoma

## SUPPLEMENTARY FIGURES

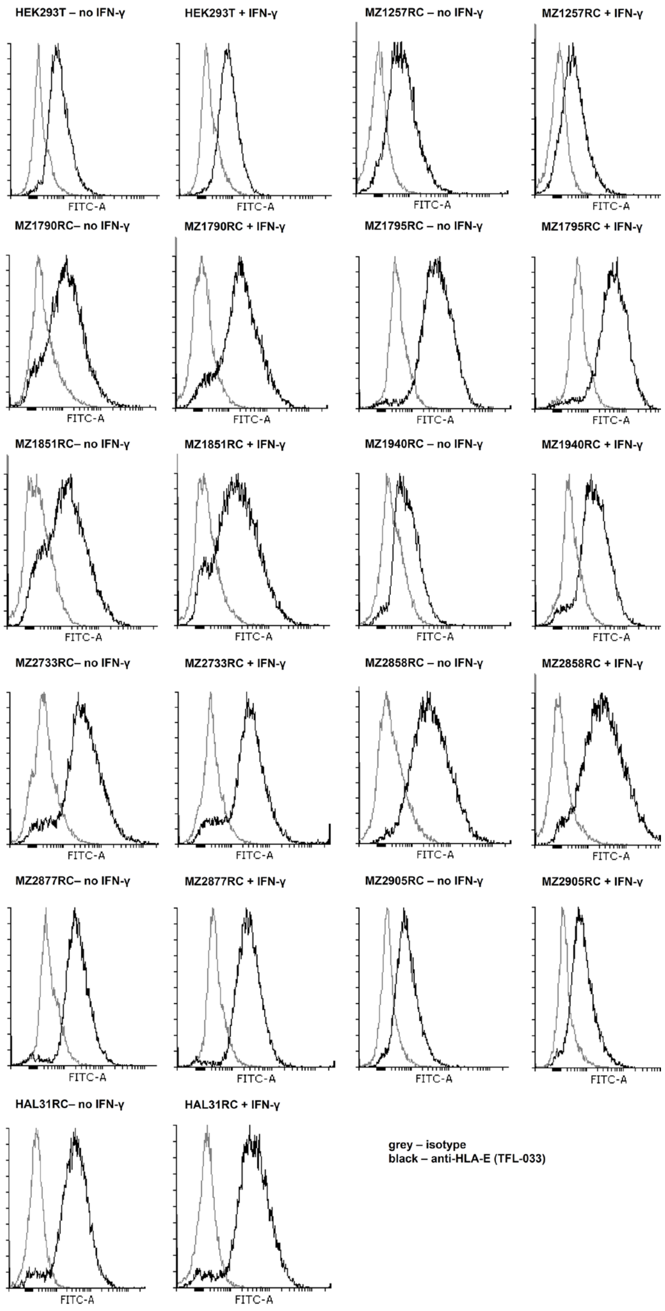


**Supplementary Figure S1: Representative histograms of intra-cellular HLA-E staining in RCC cell lines.** This Figure shows the results of the intra-cellular HLA-E expression in the applied cell lines (one biological replicate of each) +/- IFN-γ stimulation for 48 h expressed as histograms. Therefore the TFL-033 antibody was used. The mean of the three biological replicates including standard derivation and significance tests are expressed in Figure 1C.

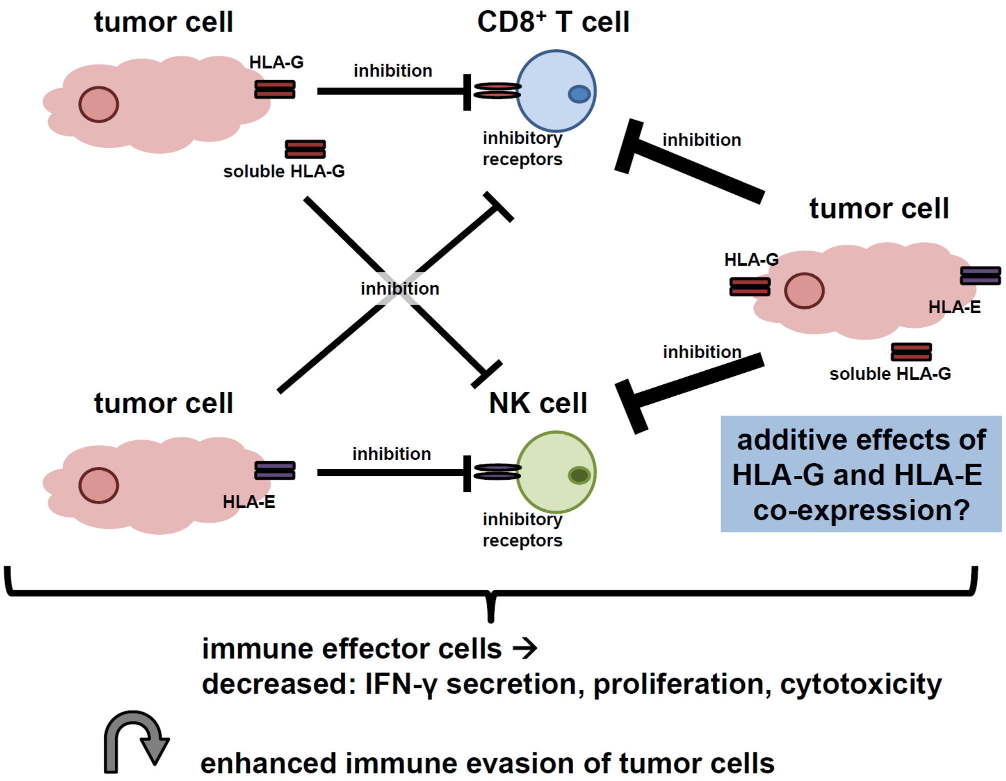


**Supplementary Figure S2: Schematic diagram.** This scheme summarizes the working hypothesis and combines also the results about the HLA-G expression the applied RCC TMA and RCC cell lines published at Jasinski-Bergner et al., 2015 [27]. HLA-G, which interacts with ILT2, ILT4 and KIR2DL4 inhibits NK activity. Additionally, HLA-E, which binds to CD94/NKG2A, -B and –C is associated to a decreased NK cell and T cell activity. However, tumors that are double positive for HLA-G and HLA-E could per se show additive effects of both immunomodulatory molecules to increase the chance of immune evasion.
